# Tachycardia and hypertension enhance tracer efflux from the spinal cord

**DOI:** 10.1186/s12987-021-00279-8

**Published:** 2021-10-26

**Authors:** Shinuo Liu, Lynne E. Bilston, Marcus A. Stoodley, Sarah J. Hemley

**Affiliations:** 1 Department of Clinical Medicine, Faculty of Medicine, Health and Human Sciences, Macquarie University, Macquarie Park, NSW 2109 Australia; 2 Neuroscience Research Australia, Prince of Wales Clinical School, University of New South Wales, Sydney, NSW 2031 Australia

**Keywords:** Tachycardia, Hypertension, Interstitial fluid, Intrathoracic pressure, Spinal cord, Respiration, Cerebrospinal fluid

## Abstract

**Background:**

Disruption of cerebrospinal fluid (CSF)/interstitial fluid (ISF) exchange in the spinal cord is likely to contribute to central nervous system (CNS) diseases that involve abnormal fluid accumulation, including spinal cord oedema and syringomyelia. However, the physiological factors that govern fluid transport in the spinal cord are poorly understood. The aims of this study were to determine the effects of cardiac pulsations and respiration on tracer signal increase, indicative of molecular movement following infusion into the spinal cord grey or white matter.

**Methods:**

In Sprague Dawley rats, physiological parameters were manipulated such that the effects of spontaneous breathing (generating alternating positive and negative intrathoracic pressures), mechanical ventilation (positive intrathoracic pressure only), tachycardia (heart atrial pacing), as well as hypertension (pharmacologically induced) were separately studied. Since fluid outflow from the spinal cord cannot be directly measured, we assessed the molecular movement of fluorescent ovalbumin (AFO-647), visualised by an increase in tracer signal, following injection into the cervicothoracic spinal grey or white matter.

**Results:**

Tachycardia and hypertension increased AFO-647 tracer efflux, while the concomitant negative and positive intrathoracic pressures generated during spontaneous breathing did not when compared to the positive-pressure ventilated controls. Following AFO-647 tracer injection into the spinal grey matter, increasing blood pressure and heart rate resulted in increased tracer movement away from the injection site compared to the hypotensive, bradycardic animals (hypertension: p = 0.05, tachycardia: p < 0.0001). Similarly, hypertension and tachycardia produced greater movement of AFO-647 tracer longitudinally along the spinal cord following injection into the spinal white matter (p < 0.0001 and p = 0.002, respectively). Tracer efflux was strongly associated with all blood vessel types.

**Conclusions:**

Arterial pulsations have profound effects on spinal cord interstitial fluid homeostasis, generating greater tracer efflux than intrathoracic pressure changes that occur over the respiratory cycle, demonstrated by increased craniocaudal CSF tracer movement in the spinal cord parenchyma.

## Background

The physiology of CSF and ISF circulation in health and disease has become an area of increasing interest. In the brain it is now recognised that turnover and clearance of these fluids are critical for circulating nutrients, waste removal, as well as regulating intracranial pressure [1]. Irregularities in the movement of ISF and CSF are now recognised to play an important role in diverse neurological pathologies, including Alzheimer’s disease, multiple sclerosis and, in the spinal cord, syringomyelia [2–4]. Despite this, there is a paucity of knowledge on the anatomical pathways and drivers of this fluid movement. This has hampered attempts to understand the pathophysiology of CSF disorders including syringomyelia, a condition where there is accumulation of fluid within the spinal cord associated with a wide range of pathologies, including spinal cord injury. There may exist a common pathway that results in an imbalance of fluid inflow and outflow, leading to fluid accumulation. In order to grasp what abnormal physiology might entail, normal physiology needs to be better understood.

Perivascular spaces are sites of great importance in CNS fluid exchange. Regional variations in the density of blood vessels may affect the susceptibility of the spinal parenchyma to fluid transport pathologies. This is supported by a previous study that demonstrated that fluorescent tracer in the grey matter was transported along vasculature structures in a radial pattern to the pial surface. In contrast, tracer from the white matter preferentially travelled longitudinally along the spinal cord [5]. An inefficient fluid and solute exchange system in either white or grey matter may result in accumulation of excitotoxic and oxidative factors and further local tissue damage.

Much of the research in perivascular transport and CNS fluid homeostasis has been performed on the brain, which has generated a growing body of literature. However, the mechanisms of fluid transport in the spinal cord remain largely unexplored. Until recently, there was little information on the pathways of normal spinal fluid outflow [5]. We have provided new insight into the effects of cyclical intrathoracic pressures on tracer movement from the subarachnoid space into spinal perivascular spaces (at least at the leptomeningeal level) (Liu et al. under review). The aim of the current study was to determine the effects of respiratory and cardiovascular parameters on movement of tracer from the spinal cord parenchyma.

## Methods

Male Sprague–Dawley rats 8–12 weeks of age and weighing 280–430 g were used. All procedures were approved by the Animal Ethics Committee at Macquarie University (Animal Research Authority Number: 2016/032) and conducted in accordance with the Australian Code of Practice for the Care and Use of Animals for Scientific Purposes.

### Surgical preparation

Animals were placed under general anaesthesia with 5% isoflurane in oxygen, then positioned supine on a heating pad and maintained under anaesthesia with 1.5–2.5% isoflurane in 0.2 L/min of oxygen. Heart rate, oxygen saturation, respiratory rate, and temperature were closely monitored by pulse oximetry (PhysioSuite®, Kent Scientific Corporation, CT, USA) and rectal thermometer connected to a homeothermic heating pad (Harvard Apparatus, Holliston, MA, USA).

The femoral artery and vein were cannulated with polyethylene catheters pre-loaded with heparinised 0.9% saline (5000 IU/L) and attached to a 3-way-tap. The arterial line was connected to a pressure transducer, enabling the continuous measurement of blood pressure. The venous line was used to administer saline and drugs as needed. A 14G endotracheal tube was then inserted into the trachea and secured in place with silk sutures. The endotracheal tube was connected to a respiratory circuit, delivering 1.5–2.5% isoflurane in oxygen. The expiratory tubing was connected to a capnometer (Capstar-100, CWE Inc., Ardmore, PA, USA), to allow end-tidal carbon dioxide (CO_2_) monitoring, and to a respiratory circuit pressure manometer made in-house, to measure relative changes in intrathoracic pressure. In the subset of rats where tachycardia was induced, a custom-made atrial pacing wire was inserted into the right external jugular vein. The pacing wire was connected to an isolated pulse stimulator (A-M Systems Inc, model 2100) [6]. At this point all physiological vital statistics were recorded continuously for the remainder of the experiment on a data acquisition interface, Power1401 (Cambridge Electronic Device, Cambridge, UK) and recorded using Spike2 software (v6. CED Ltd., Cambridge, UK). Arterial blood gas was analysed for pH, partial pressure of oxygen, and partial pressure of CO_2_ (VetStat Electrolyte Blood Gas Analyser, IDEXX Laboratories Pty. Ltd, Australia). Respiration, blood pressure and heart rate were then modulated in isolation to investigate the effect of these physiological variables on fluid flow out of the spinal cord.

### Modulation of physiological parameters

To examine the effects of changes in respiration, the conditions tested were positive intrathoracic pressure only vs positive and negative intrathoracic pressures. Animals were either allowed to breathe spontaneously, generating both negative and positive intrathoracic pressures while connected to the respiratory circuit, or a neuromuscular blockade was administered (pancuronium bromide 0.8 mg IV induction, 0.4 mg/h IV maintenance, Astra Pharmaceuticals Pty Ltd, Sydney, NSW, Australia) followed by mechanical ventilation using a small animal ventilator (Harvard 7025 Rodent Ventilator, set at a tidal volume of approximately 1.2 mL). In these animals the negative intrathoracic pressure typically generated by natural respiration was eliminated. Pancuronium bromide is known to have vagolytic effects, resulting in hypertension and tachycardia in some animals. To counteract these effects, metoprolol (10–15 mg/kg in 0.9% saline IV) was administered when necessary. The end tidal CO_2_ was maintained within a physiological range of 3.5–4.5%.

In anaesthetised spontaneously breathing rats (SB), a respiratory rate of 50–55 breaths/ min was observed, with resultant CO_2_ retention and respiratory acidosis. The respiratory rate and blood gas profile of the mechanically ventilated controls (MV) was matched to that of their spontaneous breathing counterparts. These animals also served as controls for experiments investigating the effects of heart rate and blood pressure modulation on fluid outflow. Other variables including weight, heart rate, CO_2_, circuit pressure and mean arterial pressure (MAP) were recorded. Comparison of these physiological variables is shown in Table. 1.

**Table 1:** Comparison of physiological variables

	Grey matter	White matter
Respiration: spontaneous breathing vs Mechanical ventilated controls		
Mass	ns	p = 0.03
Mean arterial pressure	ns	ns
Heart rate	ns	ns
CO_2_	ns	ns
Blood pressure: high blood pressure vs low blood pressure		
Mass	ns	0.005
Respiratory rate	ns	ns
Heart rate	0.0008	0.0005
CO_2_	ns	ns
Circuit pressure	ns	ns
Heart rate: high heart rate vs low heart rate		
Mass	ns	0.006
Respiratory rate	ns	ns
Mean arterial pressure	ns	0.004
CO_2_	0.02	0.0003
Circuit pressure	ns	ns

Physiological vital statistics were compared with two tailed Student’s t-test. The p values for the analysis are shown

*ns* non-significant

To examine the effects of blood pressure, hypertensive rats were compared to the MV group, which had an approximate MAP of 70 mmHg. Hypertension was induced by an infusion of phenylephrine, used to raise the MAP to a target of 140 mmHg (a ~ 40% increase from base line and approximately double that of controls). To prevent baroreflex compensation, and maintain heart rate, the nicotinic receptor antagonist hexamethonium was administered. All hypertensive animals were mechanically ventilated to relative hypercapnic levels (matching the MV control group).

To examine the effect of heart rate, tachycardic rats were compared to the MV control group, which had an approximate heart rate of 330 beats/min (bpm). A pacing wire (described above) was connected to an isolated pulse generator (A-M Systems Inc, model 2100). A 2 ms pulse duration and an amplitude ~ 1.0 V was used to increase heart rate to ≥ 500 bpm. Blood pressure remained stable for the duration of atrial pacing. All tachycardic animals were mechanically ventilated to a relative hypercapnic level (matching the MV group).

### Surgical procedures for investigation of tracer efflux from spinal cord

Once the desired physiological parameter was manipulated, the efflux of spinal ISF was assessed by analysing the rostrocaudal distribution and clearance of fluorescent ovalbumin (AFO-647) injected into the spinal parenchyma. The spinal grey and white matter were investigated separately. Animals were placed in the prone position, and following muscle dissection to expose the bony anatomy, a right sided hemilaminectomy at T1 was performed. A Hamilton syringe (Hamilton Company, Reno, USA) fitted with a 34G needle and positioned in a stereotaxic frame was inserted into the spinal parenchyma at a position 0.5 and 1 mm lateral to the dorsal midline vein, for the grey and white matter respectively [5]. To prevent CSF leak the dura was punctured in a single pass and cyanoacrylate glue was applied around the puncture site. A 500 nL bolus of fluorescent ovalbumin tracer, Ovalbumin Alexa-Fluor®-647 conjugate (AFO-647, Life Technologies, Victoria, Australia) was injected at a rate of 2 nL/s. The point of injection was confirmed by the presence of fluorescent microspheres FluoSpheres™ (ThermoFisher Scientific, Massachusetts, USA) (Fig. 6a). The needle was left in situ for the duration of the tracer experiment (180 min), after which the animal underwent transcardiac perfusion with 4% paraformaldehyde.

### Tissue processing and immunohistochemistry

The brain and spinal cord were dissected *en bloc* for macroscopic fluorescent imaging. The spinal cord was then segmented from C2–T4 after post-fixation and cryoprotection. Each spinal segment was embedded in Optimal Cutting Temperature Compound (Tissue-Plus™ O.C.T. Compound, Thermo Fisher Scientific, Massachusetts, USA) and frozen on dry ice. Axial sections were cut on a cryostat at 40 μm thickness and mounted onto glass slides for immunohistochemistry. To label the endothelium, slides were incubated with the primary Rat Endothelial Cell Antibody (RECA-1, Abcam, Cambridge, UK) in 4% Normal Donkey Serum (NDS), followed by the secondary antibody, anti-mouse IgG Alexa-Fluor®-488 (Molecular Probes, Life Technologies, New York, USA). Smooth muscle cells were then labelled by anti-actin, α-smooth muscle-Cy3™ antibody (SMA, Sigma-Aldrich, St. Louis, Montana). Slides were cover-slipped with fluorescent mounting medium (DAKO, NSW, Australia).

### Image acquisition

The rostrocaudal macroscopic distribution of AFO-647 along the neuraxis was captured with white-light and a single fluorescent channel (excitation wavelength 630 nm and emission wavelength 700 nm, exposure time of 4 s) using the small animal optical imaging system MS FX PRO (Bruker UK Ltd.). Images were taken from both the dorsal and ventral directions.

Spinal cord axial sections from C2–T4 were imaged with a Zeiss Axio Imager fluorescence microscope (Carl Zeiss Microimaging GmbH, Germany). Immunohistochemistry was used to image the spinal vasculature and identify vessel types. Arterioles were identified as vessels positive for RECA-1 and SMA, whereas venules and capillaries were labelled by RECA-1 only. Blood vessels that had a luminal diameter < 6.5 μm were classified as capillaries. Confocal microscopy (LSM 880, Carl Zeiss Microimaging GmbH, Germany) was used to further characterise vascular structures and the central canal.

### Image processing and analysis

In all macroscopic fluorescence images, the white-light image was used to delineate regions of interest at each spinal level from C2-T4. The tracer signal was then measured from the fluorescent image at each of these spinal levels on the dorsal and ventral surfaces. In the microscopic axial sections, the integrated density of the CSF tracer (mean pixel density multiplied by area) was calculated. The whole spinal cord, white matter, and grey matter were identified as regions of interest using the manual tracing tool in ImageJ, carefully excluding the dura and nerve roots. At least three sections were analysed per level from C2-T4, and averaged. All analysis was carried out using ImageJ software, version 1.46r [7].

### Statistical analysis

Physiological vital statistics were compared with two tailed Student’s t-test. Fluorescence intensities (integrated densities and mean pixel densities) were compared using two-way analysis of variance (ANOVA) and adjusted for multiple comparison using Bonferroni’s post-hoc tests. A p value ≤ 0.05 was considered statistically significant. All fluorescence values were expressed as mean ± standard error of the mean (SEM). All physiological parameter values were expressed as mean ± standard deviation (SD). GraphPad Prism (v7.02, GraphPad Software Inc, California) was used to perform all statistical analysis.

## Results

**Fig. 1:**
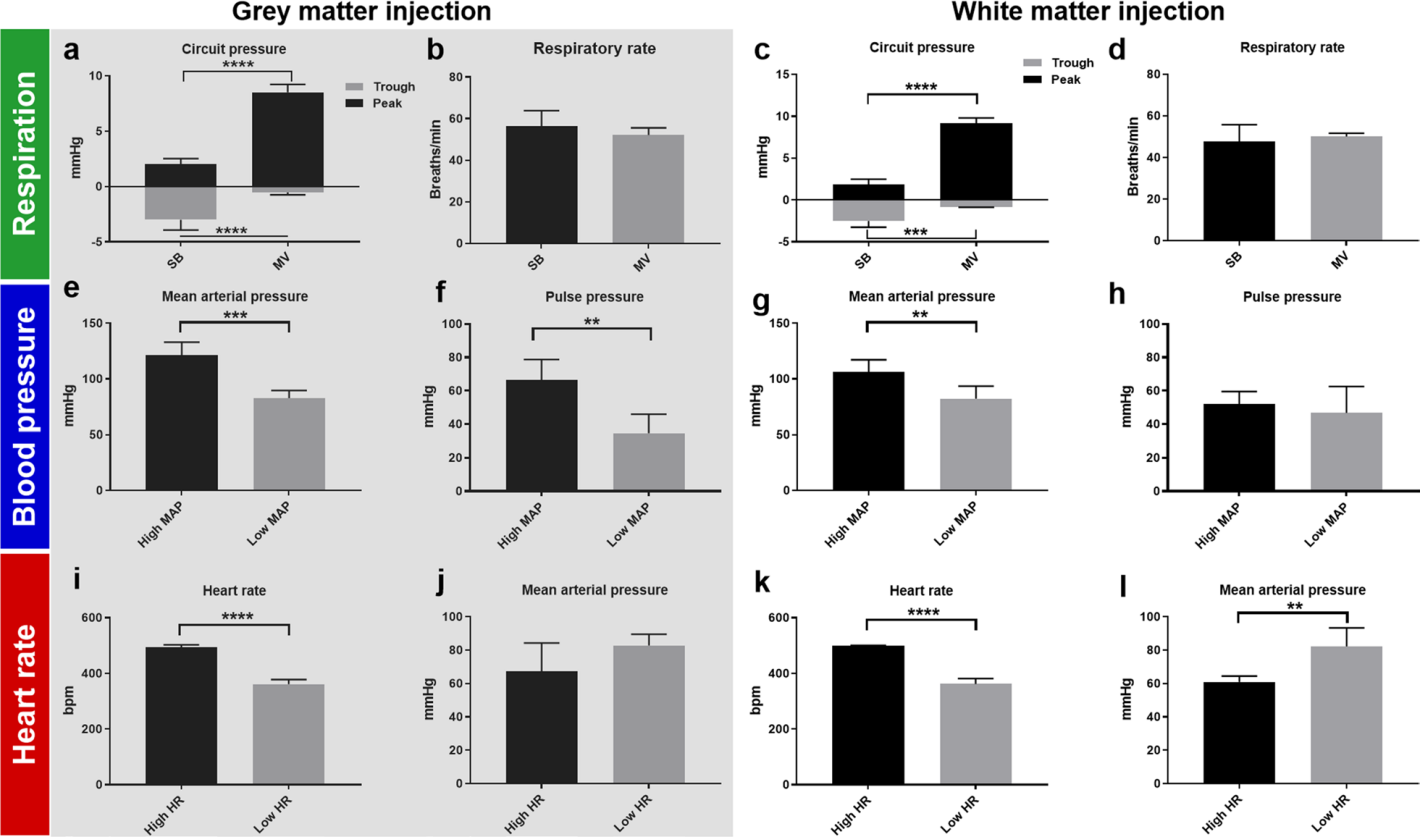
Modulation of respiration, blood pressure and heart rate. **a**–**d** To test how changes in respiration effect tracer efflux from the spinal cord, rats were allowed to either breathe spontaneously (SB) or were mechanically ventilated (MV). The peak circuit pressures (an indirect measurement of intrathoracic pressure) of MV cohorts were significantly higher than that of SB. Similarly, the trough pressures were more negative in the SB group than the MV controls. **b**, **d** SB and MV rats had similar respiratory rates (around 50 breaths/ min). **e**–**h** To test the effect of blood pressure on tracer efflux from the spinal cord, phenylephrine infusions were administered to induce hypertension. **e**, **g** significantly higher mean arterial pressures (MAP) were achieved following both white and grey matter injection. **f**, **h** Significantly higher pulse pressures were achieved in the High MAP cohort compared to the Low MAP controls following injection into the spinal grey matter. **i**–**l** To investigate the effects of heart rate on tracer efflux from the spinal cord, tachycardia was achieved by electrically pacing animals to 500 bpm. These animals had heart rates approximately 50% higher than the mechanically ventilated controls, with similar or lower blood pressures compared to the low heart rate controls, demonstrated in **j**, **l**. The graphs represent data obtained from animals that received an intraspinal injection of fluorescent ovalbumin (AFO-647) in the grey matter **a**, **b**, **e**, **f**, **i**, **j** and white matter **c**, **d**, **g**, **h**, **k**, **l** Two tailed Student’s t-test, **p < 0.01, ***p < 0.001, ****p < 0.0001. All error bars are expressed as ± SD, n = 10 rats

The impact of respiration and arterial pulsations on tracer movement in the spinal cord was assessed. The physiological parameters including intrathoracic pressure (measured indirectly via the respiratory circuit), respiratory rate, heart rate, blood pressure, partial pressure CO_2_, and arterial blood pH, of each animal were closely monitored. The variables tested are shown in Fig. 1.

### Tachycardia and hypertension increase tracer efflux from the spinal cord

#### Macroscopic quantification of AFO-647

**Fig. 2:**
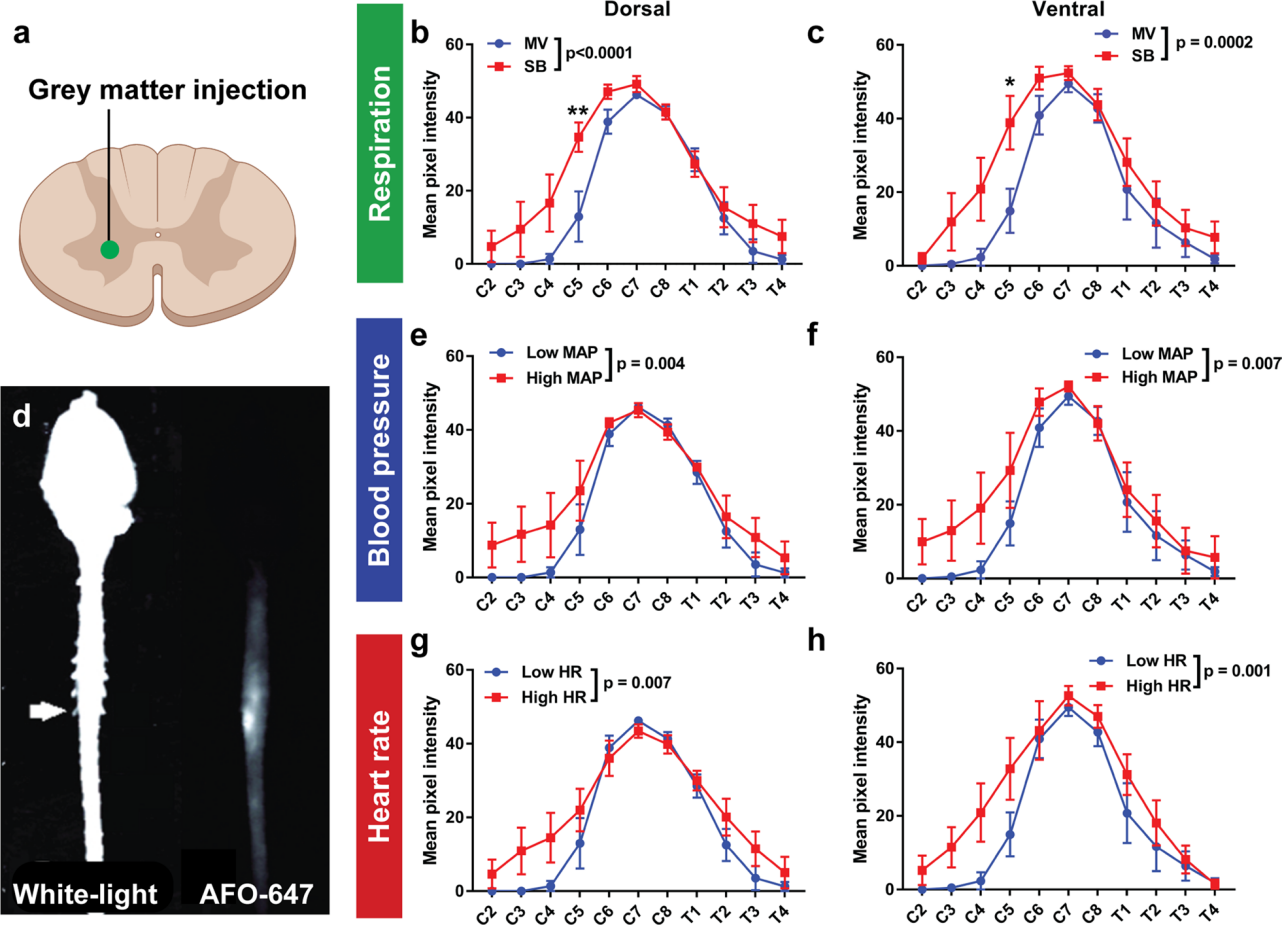
Negative intrathoracic pressure, hypertension and tachycardia increase tracer distribution along the spinal cord after grey matter injection. **a** fluorescent ovalbumin (AFO-647) was injected into the spinal grey matter. **d** Macroscopic distribution of AFO-647 in the whole neuraxis was imaged. A white-light image was produced to identify individual spinal levels and fluorescent images of the ventral and dorsal surface were taken. Macroscopic fluorescence was highest around the point of injection at C8, tapering rostrally and caudally. **b**, **c** Experiments investigating the effects of changes in respiration compared mechanically ventilated controls (MV) with spontaneous breathing (SB) animals. This tested the effect of positive intrathoracic pressures only (the MV animals were given a neuromuscular blockade to stop any diaphragmatic movement) with SB animals with both positive and negative intrathoracic pressures. **e**, **f** Experiments investigating the effects of blood pressure compared 2 groups of mechanically ventilated animals, a control group with low mean arterial pressure (Low MAP) and a group with hypertension (High MAP). **g**, **h** To test the effect of heart rate on tracer movement through the spinal cord, mechanically ventilated controls with low heart rate (HR) were compared with mechanically ventilated animals with tachycardia (High HR). SB, hypertensive and tachycardic rats all displayed significantly higher tracer intensity over both the ventral and dorsal surfaces. On post hoc analysis, the difference in tracer intensities reached significance at C5 in the respiration experiments. Two-way analysis of variance (ANOVA) post hoc Bonferroni’s *p < 0.05, **p < 0.01. All error bars are expressed as ± SEM, n = 10 rats

**Fig. 3:**
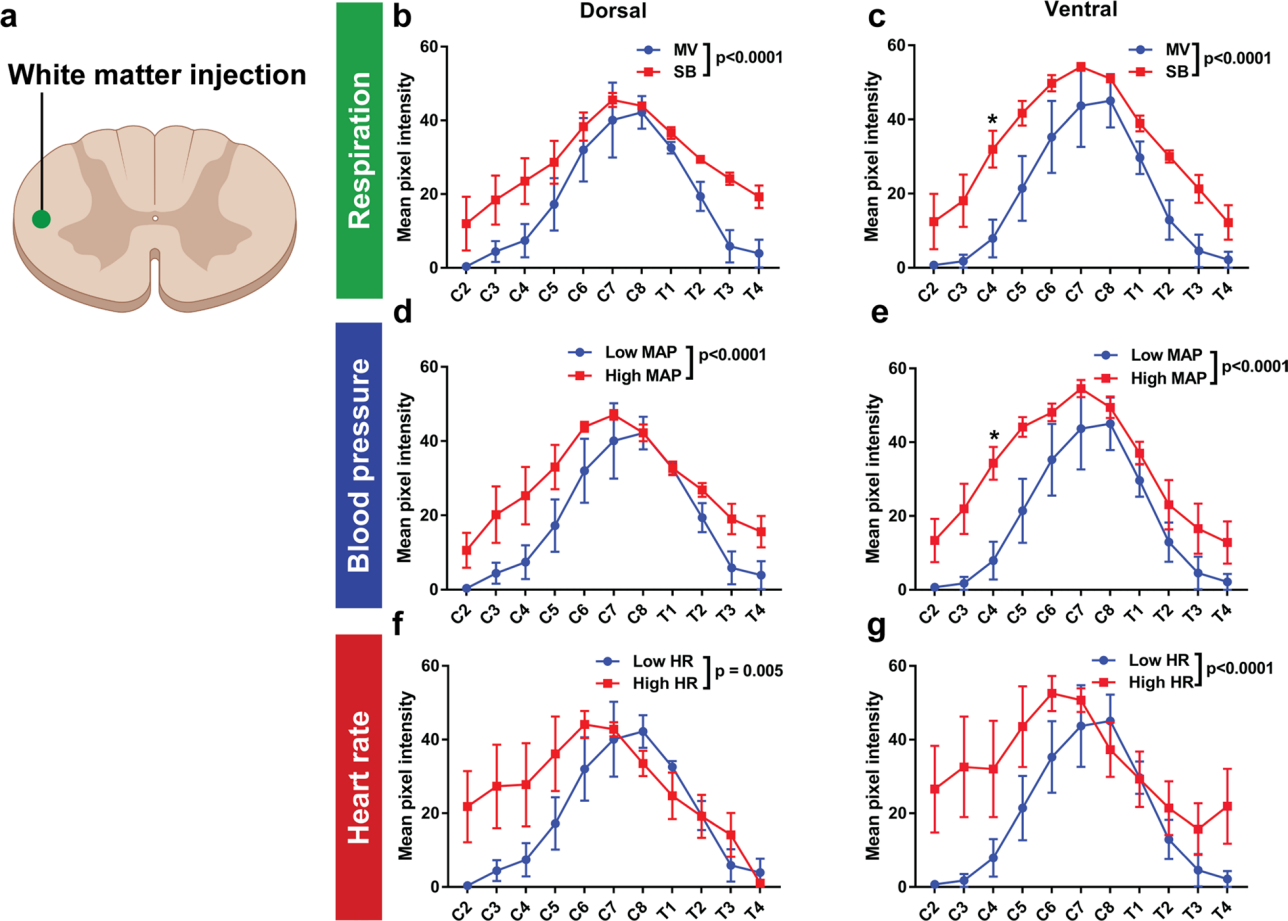
Negative intrathoracic pressure, hypertension and tachycardia increase tracer distribution along the spinal cord after white matter injection. **a** fluorescent ovalbumin (AFO-647) was injected into the spinal white matter. **b**, **c** Experiments investigating the effects of changes in respiration compared mechanically ventilated controls (MV) with spontaneous breathing (SB) animals. This tested the effect of positive intrathoracic pressures only (the MV animals were given a neuromuscular blockade to stop any diaphragmatic movement) with SB animals with both positive and negative intrathoracic pressures. **d**, **e** Experiments investigating the effects of blood pressure compared 2 groups of mechanically ventilated animals, a control group with low mean arterial pressure (Low MAP) and a group with hypertension (High MAP). **f**, **g** To test the effect of heart rate on tracer movement through the spinal cord, mechanically ventilated controls with low heart rate (HR) were compared with mechanically ventilated animals with tachycardia (High HR). SB, hypertensive and tachycardic rats all displayed significantly higher tracer intensity over both the ventral and dorsal surfaces. On post hoc analysis, the difference in tracer intensities reached significance at C4 on the ventral surface in SB and hypertensive animals. Two-way analysis of variance (ANOVA) post hoc Bonferroni’s *p < 0.05. All error bars are expressed as ± SEM, n = 10 rats

The harvested spinal cord (with intact dura) was imaged to determine the macroscopic extent of tracer distribution 180 min after intramedullary injection. Fluorescence was generally highest within one level of the injection point at C8 (Fig. 2d). Tracer signal tapered rapidly beyond that. In spontaneous breathing rats, the overall tracer intensity was significantly greater than that of ventilated controls over both dorsal and ventral surfaces, after injection into both grey and white matter (Figs. 2b, c, 3b, c). On post hoc analysis, this difference reached significance on the dorsal and ventral surface at C5 after injection into grey matter (Fig. 2b, c), and at C4 on the ventral surface after injection into the white matter (Fig. 3c).

There was also greater tracer signal overall in hypertensive (Figs. 2e, f, 3d, e) and tachycardic (Figs. 2g, h, 3f, g) animals compared with ventilated controls over both surfaces, after injection into the white and the grey matter. On post hoc analysis, there was a significant difference on the ventral surface at C4 after white matter injections (p = 0.02) in hypertensive rats (Fig. 3e).

#### Microscopic quantification of AFO-647

After injection of AFO-647 into either the spinal grey or white matter, high interstitial fluorescence intensities were observed locally. Spread of tracer was largely limited to within one level rostral and caudal to the injection point (approximately C8). The large fluorescence intensity values around the injection site obscured differences between cohorts. Therefore, the amount of tracer bound by (and including) the pial surface from C2 to C6, and from T2 to T4 was quantified. Tracer signal from the injection site (C7–T1) was selectively excluded. Fluorescence in the dura, nerve roots and within the subarachnoid space was also meticulously excluded. Limiting the analysis in this way allowed the normal fluid pathways and movement to be identified and not confounded by large amounts of tracer that may alter fluid hydrodynamics. It was also speculated that following intramedullary injection, interstitial tracer effluxed slowly from the parenchyma into the spinal subarachnoid space where it could re-enter the spinal cord interstitium at remote levels.

**Fig. 4:**
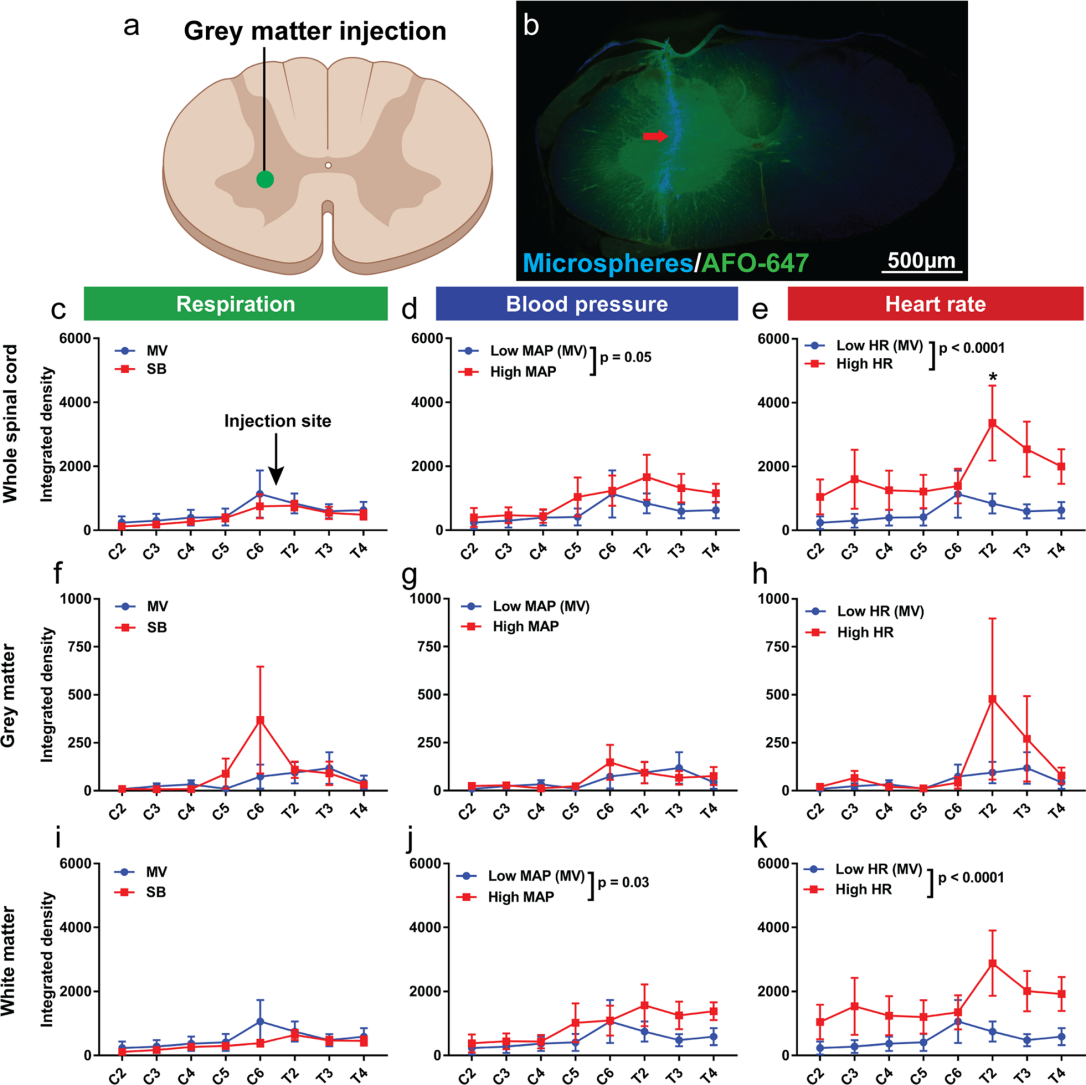
Hypertension and tachycardia increase tracer efflux from the grey matter into the white matter. Quantification of ovalbumin (AFO-647) tracer fluorescence within microscopic whole axial sections, grey matter and white matter. **a**, **b** After injection of AFO-647 into the spinal grey matter, the fluorescence intensity of redistributed tracer was measured within the grey and white matter of levels remote from the injection site (C2–6 and T2–T4). **b** The injection was verified using microspheres added to the tracer. **c**, **f**, **i** After injection of tracer there was no significant difference in whole axial section, grey and white matter fluorescence intensities between spontaneous breathing (SB) and mechanically ventilated control (MV) rats. **d**, **g**, **j** There was significantly higher fluorescence in hypertensive (High MAP) rats (compared with Low MAP controls) in the whole cord and white matter. **e**, **h**, **k** There were similar findings in tachycardic (High HR) rats compared with controls (Low HR). On post hoc analysis, the difference in fluorescence intensities reached significance at T2 in the whole spinal cord. Two-way analysis of variance (ANOVA) post hoc Bonferroni’s, *p < 0.05. All error bars are expressed as ± SEM, n = 10 rats

**Fig. 5:**
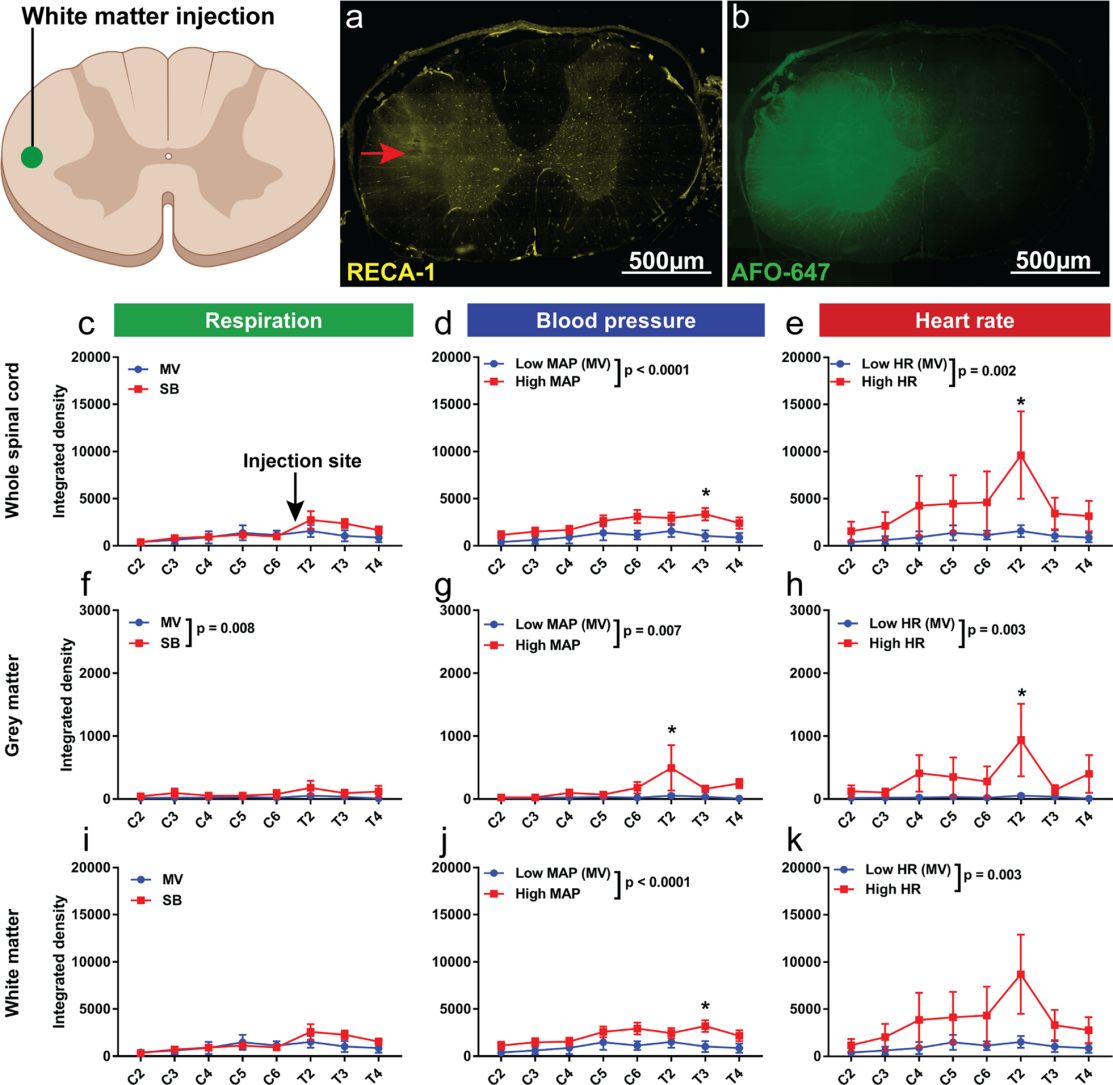
Hypertension and tachycardia have greater effect on tracer efflux from the spinal white matter than changes in respiration. Quantification of ovalbumin (AFO-647) tracer fluorescence within microscopic whole axial sections, grey matter and white matter. **a**, **b**. After injection of AFO-647 into the spinal white matter, the fluorescence intensity of redistributed tracer was measured within the grey and white matter of levels remote from the injection site (C2–6 and T2–T4). Blood vessels were labelled with an endothelial marker (RECA-1), the (*) depicts the injection site in the lateral white matter. **c**, **f**, **i** There was no difference in tracer intensity within the whole axial section or spinal white matter in spontaneous breathing (SB) rats compared with mechanically ventilated controls (MV), although significantly higher signal was detected in the grey matter. **d**, **g**, **j** There was significantly higher fluorescence in hypertensive (High MAP) rats (compared with Low MAP controls) after injection of tracer into the white matter. This was observed within both the grey and white matter. On post hoc analysis significant difference was reached at T2 in the grey matter, as well as at T3 in the white matter and within the whole axial section. **e**, **h**, **k** There were similar findings in tachycardic (High HR) rats compared with controls (Low HR), with higher tracer intensity in tachycardic rats in all parts of the spinal cord. On post hoc analysis, the difference in fluorescence intensities reached significance at T2 except in the white matter. Two-way analysis of variance (ANOVA) post hoc Bonferroni’s, *p < 0.05. All error bars are expressed as ± SEM, n = 10 rats

There was no difference in overall fluorescence within the whole spinal cord (including all its constituents) between spontaneous breathing rats and mechanically ventilated animals, after injection of tracer into the grey matter (Fig. 4c, f, i). Similar results were obtained after injection of tracer into the white matter, although higher fluorescence was observed in the grey matter of spontaneous breathing rats compared with mechanically ventilated animals (p = 0.008) (Fig. 5c, f, i).

After injection into the white matter in hypertensive rats, however, the overall fluorescence level was significantly higher than that of hypotensive controls within the whole cord (p < 0.0001), the grey matter (p = 0.007) as well as the white matter (p < 0.0001) (Fig. 5d, g, j). On post hoc analysis, these differences reached statistical significance at T3 within the whole cord (p = 0.04) and in the white matter (p = 0.05), and at T2 in the grey matter (p = 0.02). There were similar findings after tracer injection into the grey matter (Fig. 4d, g, j). In hypertensive rats, fluorescence was significantly higher than hypotensive controls within the whole spinal cord (p = 0.05) and white matter (p = 0.03), but no difference was detected in the grey matter.

After injection into the white matter in tachycardic rats (Fig. 5e, h, k), the fluorescence intensity was higher compared with bradycardic controls within the whole cord, the grey matter and the white matter (p = 0.002, 0.003 and 0.003, respectively). On post hoc analysis, a significant difference was reached at T2 in the grey matter and whole spinal cord (p = 0.04 and 0.03 respectively). After injection into the grey matter of tachycardic rats (Fig. 4e, h, k), there was increased tracer signal within the whole cord and the white matter (both p < 0.0001) compared with bradycardic controls. No difference, however, was observed in the grey matter. On post hoc analysis, significant difference was reached at T2 within the whole cord after injection (p = 0.03).

#### Tracer efflux occurs via perivascular pathways

**Fig. 6:**
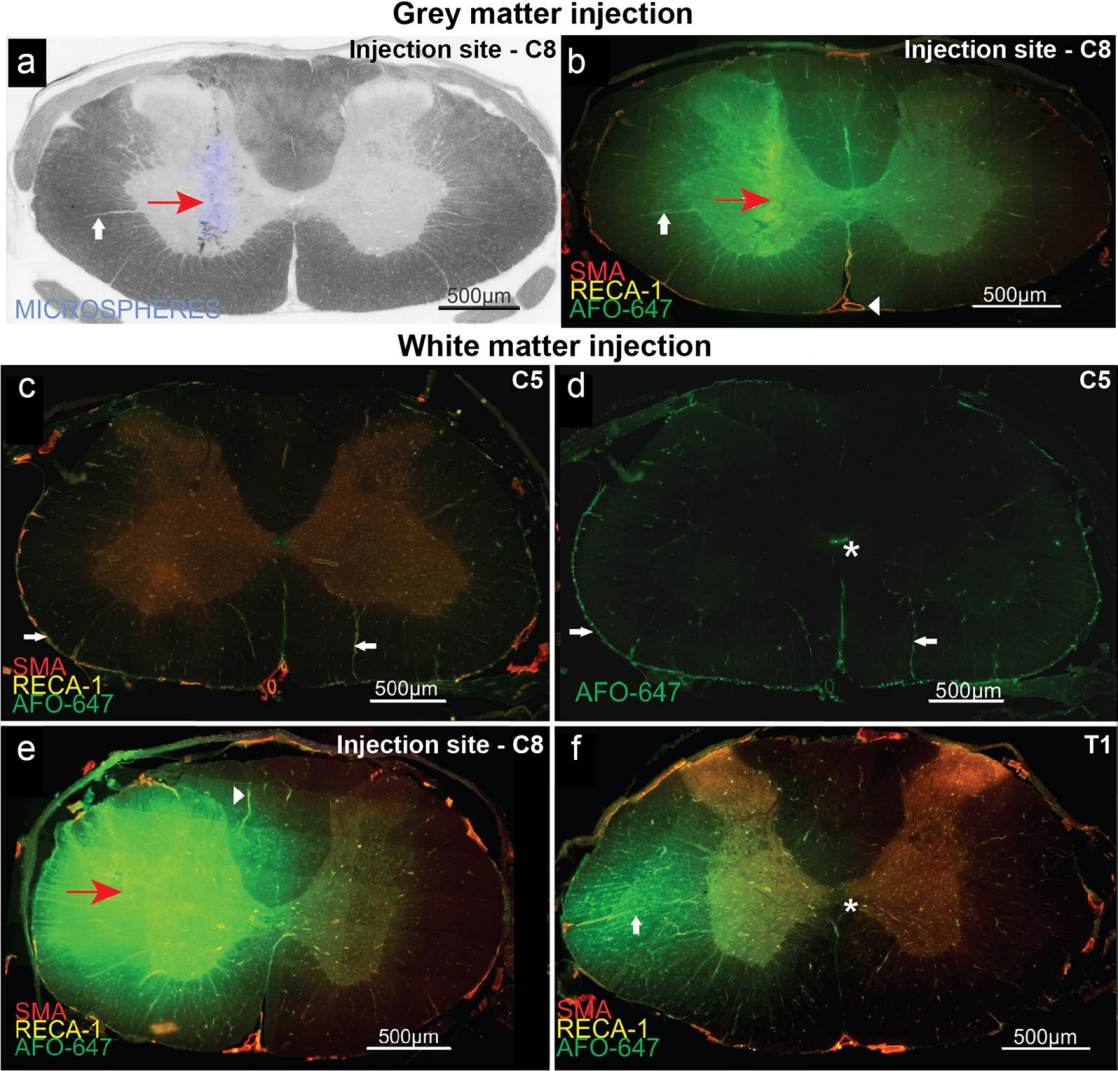
Tracer efflux from the grey and white matter follow similar pathways. Qualitative analysis of redistributed fluorescent ovalbumin (AFO-647) after injection into the spinal interstitium. **a**, **b** The spinal grey matter was cannulated by a fine needle (at approximately C8). Inert fluorescent microspheres (marked by red arrow) combined with the AFO-647 tracer confirmed the injection point. After grey matter injections, there was radial redistribution of tracer locally into the surrounding white matter and into the contralateral hemicord. There was selective concentration of tracer around radially projecting blood vessels (up arrow) that end ultimately on the pial surface. Extramedullary vessels, such as the anterior spinal artery (left arrowhead) were also labelled by tracer. **c**, **d** Three spinal levels rostral to the white matter injection site (approximately C5), tracer was found circumferentially on the pial surface (right arrow) and around penetrating radial blood vessels (left arrow). Note that subpial tracer was absent. **e** After white matter injections (location marked by red arrow), there was local radial spread of tracer into the grey matter and nearby subpial white matter. Less of the contralateral hemicord was involved. There was accumulation of tracer around radially projecting blood vessels (right arrowhead). **f** Just caudal to the white matter injection site (approximately T1), intramedullary tracer signal was delimited by the lateral white matter tracts (up arrow). **d**, **f** Note AFO-647 tracer deposition around the central canal (*) rostral and caudal to the injection site. This pattern of tracer distribution is highly suggestive of interstitially injected AFO-647 effluxing into the subarachnoid space, redistributing rostrocaudally and re-entering the cord parenchyma via perivascular spaces. Smooth muscle cells were labelled with SMA, endothelial cells by RECA-1

Here, a qualitative study of interstitially delivered AFO-647 relative to spinal microanatomical structures was undertaken. After grey matter injections, there was radial distribution of tracer from the point of injection towards the pial surface and into the contralateral hemicord (Fig. 6b). The fluorescence intensity attenuated with distance from the injection point, such that subpial fluorescence was absent. Rostral and caudal to the injection site, tracer was largely found within the grey matter, although it readily crossed the white/grey junction. After white matter injections, tracer crossed from the white matter into the grey matter and into the contralateral hemicord around the site of injection (Fig. 6e). However, tracer was largely confined to the lateral column rostrocaudally (Fig. 6f).

**Fig. 7:**
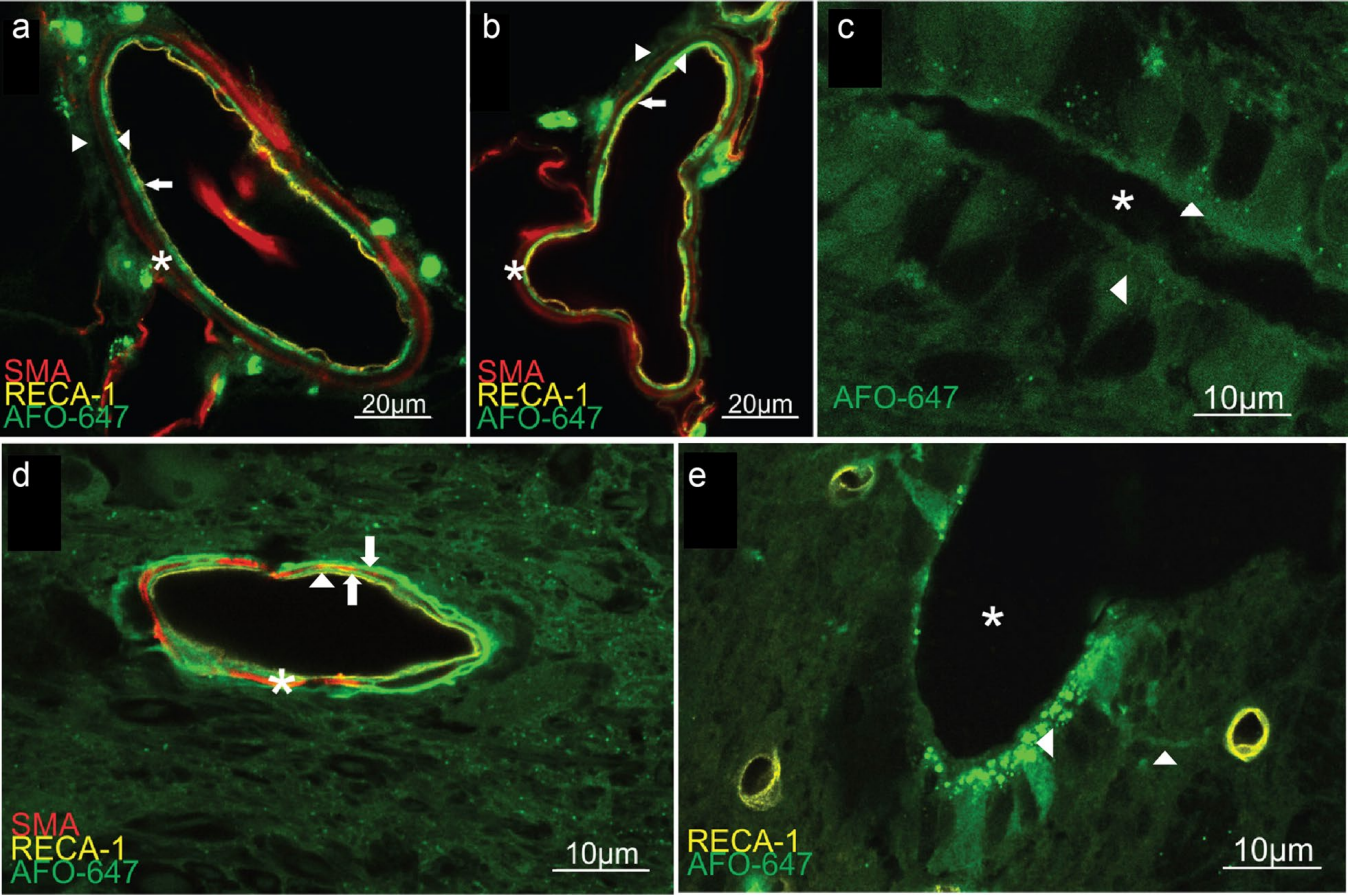
Fluorescent ovalbumin (AFO-647) injected into the spinal interstitium labels blood vessels and central canal. **a**, **b**, **d** In both extramedullary and intramedullary arteries and arterioles, tracer deposited distinctly in multiple layers (confocal microscopy, × 100 magnification). AFO-647 was found external to, and within the tunica media (marked by *) of the anterior spinal artery, **a**, grey matter arteriole, **d**, and arterial vasocorona, **b**. Arrows mark the yellow endothelium, while the layers of green AFO-647 are marked by oppositely pointing sets of arrow heads and arrows. **d** Note the delineation of neuronal nuclei and the tortuous spinal extracellular space by AFO-647. **c**, **e** The central canal at × 100 magnification on confocal microscopy. The left arrow head in **e** and the up arrow head in **c** emphasise the heterogeneous, speckled deposition of tracer favouring the luminal aspect of the ependymal lining (* is the lumen of the central canal). Tracer also deposited around ependymal nuclei (marked by left arrowhead in **c**). The up arrow head in **e** points to a serpiginous trail of tracer, suggestive of privileged pathways between the subependymal vasculature and the central canal. Smooth muscle cells were labelled with SMA, endothelial cells by RECA-1

At the level of the injection site of all rats, tracer selectively deposited around radially projecting arterioles and venules (Fig. 6b–f). These appeared to be privileged pathways for tracer efflux towards the pia. The anterior spinal artery and other extramedullary vessels were invariably labelled even though subpial tracer was not present (Figs. 6b, 7a).

At spinal levels rostral or caudal to the injection site, AFO-647 was detected circumferentially on the pial surface and around extramedullary vessels (Fig. 6d). Rostral or caudal to the injection site, interstitial fluorescence was limited to the perivascular spaces of radially projecting arterioles and venules (Fig. 6d), and not present in the parenchyma. This was highly suggestive of tracer effusing from the injection site to the subarachnoid space, and then redistributing back into the spinal parenchyma at distant levels, via perivascular routes.

Confocal microscopy was used to identify the precise location of tracer deposition. In both extramedullary and intramedullary arteries and arterioles, distinct layers of AFO-647 interdigitated with the layers of the tunica media (Fig. 7a, b, d). Tracer was found external to, as well as within, the smooth muscle wall. AFO-647 was found around the RECA-1 labelled endothelial layer of intramedullary venules and capillaries as well as the venules within the ventral median sulcus. The location of tracer confirmed that pathways of perivascular efflux are similar to inflow [8].

After both white and grey matter injections, there was ependymal deposition of tracer around the central canal in approximately half (45–55%) of all spinal levels, with slightly higher rates of deposition in tachycardic (56%) and hypertensive (62%) animals. These data are summarised in Table 2. Confocal microscopy revealed continuous bands of tracer that appeared to bridge microvessels and the heterogeneously labelled central canal (Fig. 7c, e). This is evidence of possible specialised pathways between subependymal perivascular spaces and the abluminal aspect of the ependymal layer (Fig. 7e).

**Table 2:** CSF tracer in the central canal

	Grey matter injection (%)	White matter injection (%)
MV control	45	45
Spontaneous breathing	55	47
Hypertensive	51	62
Tachycardic	45	56

The percentage (%) of spinal levels with CSF tracer (AFO-647) deposited in the central canal following injection into the white and grey matter for each cohort of animals

*MV* mechanically ventilated

## Discussion

Hypertension and tachycardia appear to increase tracer efflux from the cord into the subarachnoid space. Intrathoracic pressure had less of an effect on the distribution of tracer away from the injection site (Figs. 4, 5). However, there was increased tracer signal macroscopically in the craniocaudal axis in spontaneous breathing rats compared with mechanically ventilated animals. This could be explained by the fact that, unlike the spinal dural sac which is directly exposed to shifts in epidural venous plexus pressures (brought about by changes in intrathoracic pressure) and readily enlarges or collapses in response to these pressure gradients [9], the spinal cord itself may be protected from these fluctuating pressure changes. The amount of solute egress from the cord is therefore minimally affected by changes in respiration. However, any tracer that is drained into the subarachnoid space from the cord parenchyma is mixed more efficaciously and distributed further along the spinal subarachnoid space, possibly resulting in higher fluorescence intensities on the surface of the spinal cord. Moreover, the amount of tracer that dissipated into the subarachnoid space may be too small for a difference in tracer signal (between the spontaneous breathing and control groups) to be detected when it is subsequently redistributed back into cord parenchyma at remote spinal levels.

In our previous study of molecular transport from the rat spinal cord, tracer redistribution was assessed at 20 and 60 min after delivery into the parenchyma [5]. Fluorescence intensity did change over time, suggesting that tracer spread was not artefactual due to the tracer infusion, but indicative of solute transport. Nevertheless, interstitial spread was limited to within one level caudal and rostral to the injection point. Here, we have demonstrated that even 180 min after delivery (the upper limit of length of time rats could be kept haemodynamically stable under altered physiology), endogenous tracer movement remained limited to adjacent spinal levels. Tracer injected into the grey matter demonstrated radial redistribution outwards from the injection point. Delivered into the white matter, AFO-647 was largely confined within the parallel myelinated tracts. This is consistent with previous distribution patterns with isotropic distribution in the grey matter and anisotropic distribution in the white matter [5]. These results suggest that diffusion governs endogenous spinal solute and fluid transport, although advanced techniques such as integrated optical imaging and real-time iontophoresis [10] will be required to confirm this. There is mounting in silico evidence that convective flow, as suggested by the glymphatic theory, is implausible in the extracellular space of the CNS [11]. Concerns about the interpretation of ex vivo preparations in efflux studies should be addressed by real-time in vivo imaging of injected extracellular space tracers. Arbel-Ornath et al*.* [12] employed two-photon intravital microscopy to track fluorescent dextrans injected into murine brain. A rapid co-localisation to the arterial basement membrane of the perivascular space was reported. No tracers were detected around venous structures. Biexponential reduction of tracer over 30 min was interpreted by the investigators as evidence of bulk flow in the perivascular space. However, in these experiments the dura was opened, compromising the hydraulic integrity of the system. No equivalent investigation has been undertaken in the spine.

Whether CNS solutes are cleared primarily into lymphatics of the dura and large blood vessels, or into the subarachnoid space is still unclear [13, 14]. There are likely differences among animal species and variations with age and pathological conditions. Substantial accumulation of tracer around the pia, with subsequent perivascular re-entry of tracer into the parenchyma at spinal levels distant to the injection was not observed in our previous study, up to 60 min after injection [5]. The results of the current study, however, indicate that transport of tracer from the spinal cord is at least partially to the subarachnoid space.

Previously, Hadaczek et al. [15] showed adrenaline-induced hypertension and tachycardia promoted apparent bulk flow of different sized macromolecules through the extracellular space of rat brain. In a murine model of Alzheimer’s Disease exposed to cerebral hypoperfusion (and presumably low blood pressure), mural Aβ was observed to accumulate in leptomeningeal vessels, reflecting reduced solute drainage [16]. The results from our study support a similar role of blood pressure and heart rate in the spinal cord, with increases to either physiological value promoting tracer movement. Mathematical modelling also supports cardiac pulsations driving this flow [17]. In contrast, another study investigating CSF tracer influx into the brain reported that decreased heart rate correlated with improved molecular clearance from mouse brain [18]. It is interesting to note that a recent study by our group looking at the same physiological factors found that the alternating positive and negative intrathoracic pressures that occur during spontaneous breathing had a greater effect on CSF flow from the subarachnoid space into the spinal cord when compared with mechanically ventilated controls with continuous positive intrathoracic pressure. It is possible that the physiological factors that govern tracer influx exert different effects on tracer efflux.

In the current experiments, tracer accumulated in distinct layers both internal and external to the smooth muscle layer of intramedullary and extramedullary arterioles and arteries (Fig. 7). Around veins, venules, and capillaries, AFO-647 was deposited in close proximity to the endothelium. Thus, all blood vessel types have been implicated in spinal outflow, similar to findings from recent spinal inflow studies [8]. These findings recapitulate results from earlier work on molecular transport pathways in the normal spinal cord [5]. Dedicated ultrastructural studies will be required to clarify the precise anatomical relationships of the tracer and the various compartments of the perivascular space. However, these results are supportive of the role of vascular basement membrane(s), as well as the compartments between the glia limitans and pial sheath, or the adventitia, in mediating solute clearance. It should be noted that findings from ex vivo preparations should be cautiously interpreted as there is evidence that tracer deposition in some regions may be artefactual, induced either by changes that occur upon death or during the process of perfusion and fixation, which can substantially alter the structure of the perivascular spaces and tissues [13, 19]. Nevertheless, it is intriguing that pathways of influx and efflux appear shared. This raises the possibility that there is bidirectional, to-and-fro mixing of fluid in the perivascular space that is able to rapidly distribute solutes. The direction of tracer redistribution depends, therefore, on whether it is injected into the subarachnoid space (inwards, towards the central canal) or into the parenchyma (outwards, towards the pial surface) [20, 21]. It is possible that during inspiration, associated with large magnitude CSF pulse waves in the subarachnoid space, CSF is driven into the spinal cord parenchyma along perivenous and periarterial spaces. Within the spinal cord however, increased blood vessel pulsatility occurring with increases in blood pressure and heart rate, drive more fluid transport through the interstitium, enhancing efflux of solutes and fluid from the spinal cord into the subarachnoid space. In the current study, higher MAP/pulse pressure and heart rates were associated with greater endogenous tracer deposition remote from the delivery site, indicating greater efflux locally from the point of injection. Arterial pulsations, therefore, promote molecular transport in the spinal cord.

The role of the central canal in CSF and molecular transport is largely unknown. Milhorat and colleagues [22] had previously ascribed the central canal with a “sink” function for solute and metabolites. Spontaneous breathing, tachycardia and hypertension did not appear to have major effects on drainage into this compartment from the extracellular space. More than two decades ago, Cifuentes et al*.* [23] proposed the possibility of bidirectional fluid transport between the central canal and the subarachnoid space via perivascular spaces, particularly via peri-arterial pathways of the central branches of the anterior spinal artery, consistent with our findings. Contiguous bands of tracer were detected between the subependymal microvasculature and the central canal ependymal cells. To confirm and further examine this putative pathway, the next step is to fluorescently label ependymal cells (such as with F-actin [24]) in future intravital studies of influx and efflux. Tanycytes and complex ependymal basement membranes (labyrinths) are thought to subserve this putative connection between CSF, ISF and the central canal [25]. The role of the central canal in fluid exchange may not be as important in humans as there is progressive atresia of this structure with age [26].

A comprehensive model of spinal fluid transport that consolidates the findings from these experiments, as well as data from previous laboratory and computational studies, may still be out of reach. There is, however, evidence that the same anatomical pathways subserve influx and efflux, so it is reasonable to deduce that the two processes occur simultaneously and may be subject to the same physiological drivers. We have provided evidence here that tachycardia and hypertension enhance movement of tracers injected into the spinal cord parenchyma. However, this has not necessarily resulted in increased overall solute drainage. An interplay of factors—such as the width of the perivascular space as dictated by the phase difference of the arterial wave with CSF pulse wave [27], the stiffness of the arterial wall, and the opposing forces of influx and efflux which appear to occur along similar pathways—may ultimately determine the net direction and magnitude of fluid and solute exchange as the extremes of physiology are approached. Moreover, the role of the central canal in mediating intramedullary drainage, and local microanatomical geometries in the subarachnoid space at different spinal levels add further layers of complexity. Simple tracer experiments in animal models, while invaluable in our attempts to elucidate the basic mechanisms of fluid transport, cannot capture fully the driving forces governing a dynamic process.

The perivascular space has been recognized as a site of paramount importance in mediating spinal fluid exchange. After injection of AFO-647 into the spinal parenchyma, there was selective accumulation of tracer around radially projecting intramedullary as well as extramedullary blood vessels. There was a contrasting lack of subpial fluorescence, suggesting that solutes could access preferential routes for efflux from the interstitium. With time, it was apparent that tracer drained at least partially into the spinal subarachnoid space, redistributing over the pial surface distant to the injection site. Moreover, higher MAP/pulse pressure and heart rates were associated with more endogenous tracer deposition remote from the delivery site, indicating greater transport locally from the point of injection. Arterial pulsations, therefore, promote spinal efflux.

### Limitations

Caveats of the techniques used in this study need to be highlighted. Firstly, we cannot exclude the possibility that injecting 500 nL of tracer into the spinal parenchyma could cause overloading of the system. To minimise this risk, we have chosen a volume that is comparable to or lower than similar studies carried out in mouse brain [2, 28–31]. Secondly, although the complex experimental techniques were designed to modulate a single physiological parameter and measures were taken to maintain all other variables, it is possible that sympathomimetic medications such as phenylephrine can induce vasoconstriction or disturbance of spinal autoregulation. These alterations are difficult to quantify. In the current study measures were taken to mitigate changes to the integrity of the intrathecal sac. The needle was inserted with a single-pass (the dura was not opened prior to needle insertion) and cyanoacrylate glue was placed around the needle. The needle was also left in place for the duration of the experiment prior to perfusion. However, it has to be acknowledged that piercing the dura may impact the pressure within the dural sac and withdrawing the needle at the completion of the experiment just prior to perfusion-fixation would cause CSF leak and altered fluid flow within the spinal cord itself. As mentioned previously, another limitation of ex vivo experiments are the possible post-mortem ultrastructural changes that occur after cardiorespiratory arrest and fixation with aldehydes that may influence the location of the tracer and may not represent the distribution in living animals. While intravital imaging has allowed for some of these flaws to be overcome, there are still unresolved technical challenges in spinal in vivo imaging that prevent analysis of fluid and solute transport at the microscopic level. The much deeper field of visualisation is just one of these barriers.

### Clinical implications

There are no effective medical treatments for the deleterious secondary effects of spinal cord injury or the subsequent formation of fluid-filled cysts (syringomyelia). These effects are thought to be mediated by the release of excitotoxic factors that further potentiate local damage after primary injury [32]. Understanding the factors that drive fluid efflux from the spinal cord may provide an opportunity to enhance the removal of excess fluid, and harmful proteins, amino acids and other molecules.

## Conclusions

Molecular transport from the spinal cord into the subarachnoid space is increased in hypertension and tachycardia. Elimination of negative intrathoracic pressure has little effect on tracer clearance from the spinal cord parenchyma. The route of efflux appears to involve periarterial, perivenous and pericapillary pathways.

## Data Availability

The datasets supporting the conclusions of this article are available from the corresponding author on reasonable request.

## Abbreviations

ANOVA: Analysis of variance
AFO-647: Ovalbumin Alexa-Fluor®-647 conjugate
bpm: Beats per minute
CNS: Central nervous system
CSF: Cerebrospinal fluid
HR: Heart rate
ISF: Interstitial fluid
MAP: Mean arterial pressure
MV: Mechanically ventilated
NDS: Normal donkey serum
RECA-1: Rat endothelial cell antigen
SB: Spontaneous breathing
SD: Standard deviation
SEM: Standard error of the mean
SMA: Smooth muscle antibody
